# Bridging Molecular and Clinical Sciences to Achieve the Best Treatment of *Enterococcus faecalis* Endocarditis

**DOI:** 10.3390/microorganisms11102604

**Published:** 2023-10-21

**Authors:** Francesco Nappi, Sanjeet Singh Avtaar Singh, Vikram Jitendra, Antonio Fiore

**Affiliations:** 1 Department of Cardiac Surgery, Centre Cardiologique du Nord, 93200 Saint-Denis, France; 2 Department of Cardiothoracic Surgery, Royal Infirmary of Edinburgh, Edinburgh EH16 4SA, UK; 3 Department of Cardiothoracic Surgery, Aberdeen Royal Infirmary, Aberdeen AB25 2ZN, UK; vikram.jitendra@nhs.scot; 4 Department of Cardiac Surgery, Hôpitaux Universitaires Henri Mondor, Assistance Publique-Hôpitaux de Paris, 94000 Creteil, France; fioreant7@yahoo.com

**Keywords:** infective endocarditis, *Enterococcus faecalis*, *Enterococcus faecalis* pili, biofilm, transcatheter aortic-valve implantation, aortic allograft

## Abstract

*Enterococcus faecalis* (*E. faecalis*) is a commensal bacterium that causes various infections in surgical sites, the urinary tract, and blood. The bacterium is becoming a significant concern because it tends to affect the elderly population, which has a high prevalence of undiagnosed degenerative valvular disease and is often subjected to invasive procedures and implanted medical devices. The bacterium’s actions are influenced by specific characteristics like pili activity and biofilm formation. This resistance significantly impedes the effectiveness of numerous antibiotic therapies, particularly in cases of endocarditis. While current guidelines recommend antimicrobial therapy, the emergence of resistant strains has introduced complexity in managing these patients, especially with the increasing use of transcatheter therapies for those who are not suitable for surgery. Presentations of the condition are often varied and associated with generalised symptoms, which may pose a diagnostic challenge. We share our encounter with a case study that concerns an octogenarian who had a TAVI valve and developed endocarditis. We also conducted a literature review to identify the essential treatment algorithms for such cases.

## 1. Introduction

*Enterococcus faecalis* is a bacterium commonly found in the human gastrointestinal and biliary tracts. Its pathogenic effect is responsible for a significant proportion of surgical-site, urinary-tract, and bloodstream infections. Similarly, Group D streptococci (e.g., *Streptococcus gallolyticus*, *Streptococcus bovis*), which have been reclassified as *Enterococci* spp., are recognised for their ability to facilitate infective endocarditis (IE) linked with gastrointestinal- and urogenital-tract disorders that utilise the portal venous system as a means of entrance [1,2,3,4,5,6]. Studies in molecular biology and clinical therapeutics supporting enterococcal IE have indicated that the pili’s role is relevant to greater bacterial aggression due to their biogenesis, host immune response, and resistance to antimicrobial therapy [7,8,9,10,11,12]. Research has shown that F pili have three key functions in bacterial mating: firstly, they initiate contact between mating pairs; secondly, they facilitate the transfer of genetic material; and lastly, they draw the mating cells into close contact, which boosts the fertility of the bacterial union [13,14]. This could partly clarify the involvement of *E. faecalis* as one of the causative pathogens of bacterial IE, which is responsible for high mortality rates and severe complications such as exacerbating congestive heart failure, glomerulonephritis, and septic embolism.

Current guidelines suggest treating enterococcal endocarditis with a combination of antimicrobials immediately after diagnosis in order to manage the infection medically. However, worries regarding the emergence of bacteria resistant to multiple antibiotics, including strains of enterococcus that are resistant to vancomycin, have created uncertainty around the best guideline-directed medical therapy (GDMT) by widening an increasing gap [15,16,17,18]. There is substantial epidemiological evidence that more than 20% of enterococci isolated from intensive care unit patients’ infections are resistant to vancomycin. This is supported by several studies [8,9,16].

The occurrence of IE caused by *E. faecalis* affects one third of patients over 70 years old [19,20,21,22,23], with projections indicating increasing frequency in the future due to longer life expectancy [22,23]. The highest rise in the incidence of IE worldwide is among the elderly population, with elderly patients at 4.6 times higher risk than the general population [19,23]. Factors that contribute to the promotion of IE include the high frequency of undiagnosed degenerative valvular disease, as well as an increase in invasive procedures and implanted medical devices. These factors may also impact the outcome of IE in elderly patients, who experience significantly higher morbidity and mortality rates compared to younger adults. However, international guidelines for treating IE in elderly patients lack recommendations due to limited evaluation, which hinders precise management guidance [24].

The risk of infection reoccurring after valve replacement for IE is still worrying. Therefore, there has been a long-standing debate about the best substitute for a valve in this case [2,6,17,18,24]. According to surgical dogma, autologous or allogeneic tissue is the preferred choice over synthetic materials in an infected field that contains 60% gram-positive cocci (GPC). Given the reluctance to use foreign artificial materials, such as mechanical xenograft valve prosthetics or conventional stents, cardiac surgeons have preferred the use of allogeneic tissue. This approach has been particularly effective in cases of prosthetic-valve endocarditis (PVE) and other complex and aggressive cardiac injuries, such as evolving root abscess and invasion of the intervalvular fibrosa causing bivalvular involvement (both aortic and mitral valves) and a higher risk of fistulisation in the left atrium. Reported benefits have been significant [2,6,24].

A collaborative effort through multidisciplinary research is urgently needed to identify new therapies or preventive measures that can disrupt the pathogenetic mechanisms responsible for enterococcal endocarditis. This requires clear communication and consistent use of technical terms and units of measurement. Therefore, this review aims to provide an overview of the pathogenetic mechanisms that contribute to IE caused by *E. faecalis*. Despite antimicrobial therapies, this condition can ultimately require surgery due to ineffective treatments.

The review proceeds after a case study by the IE multidisciplinary team on a complex clinical case of *E. faecalis*-induced IE to guide clinical therapeutics. We assert that the presented data in this summary may establish a foundation for comprehending the development of IE- and biofilm-associated pili in the colonisation of cardiac structures. Furthermore, we anticipate that the growing evidence will aid microbiologists and healthcare professionals including family doctors, internists, cardiologists, and cardiac surgeons in facilitating discussions with patients regarding the risks and prospects of cardiac involvement arising from *enterococcal* spp. infections.

## 2. Case Description

An 86-year-old man with a heart murmur was admitted to the emergency department with difficulty breathing, fever up to 39 °C, reduced level of alertness, and difficulty speaking clearly. A month earlier, the general practitioner diagnosed him with a non-specific “inflammatory syndrome” when he reported fever and suspected pneumonia. Antibiotics were administered empirically, and his fever improved. The patient regularly attended scheduled checkups due to aortic-valve stenosis associated with non-insulin-dependent diabetes and hypertension. Four months prior to being admitted to hospital, a self-expandable transcatheter aortic-valve implantation (TAVI) was successfully carried out via transfemoral means for severe symptomatic aortic stenosis, without a clear diagnosis of IE.

**Figure 1:**
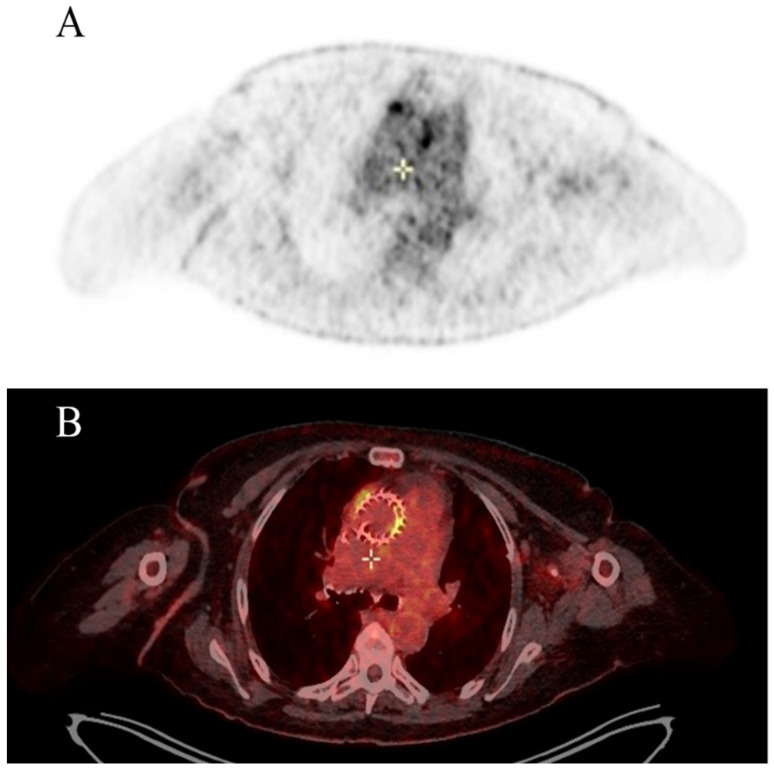
Pre-operative FDG-PET/CT imaging assessment with multifocal uptake of prosthetic transcatheter valve. (**A**) attenuation-corrected FDG-PET, axial slice; (**B**) fused attenuation-corrected FDG-PET/CT, axial slice. Abbreviation; FDG-PET/CT, fluorodeoxyglucose positron emission tomography/computed tomography.

**Figure 2:**
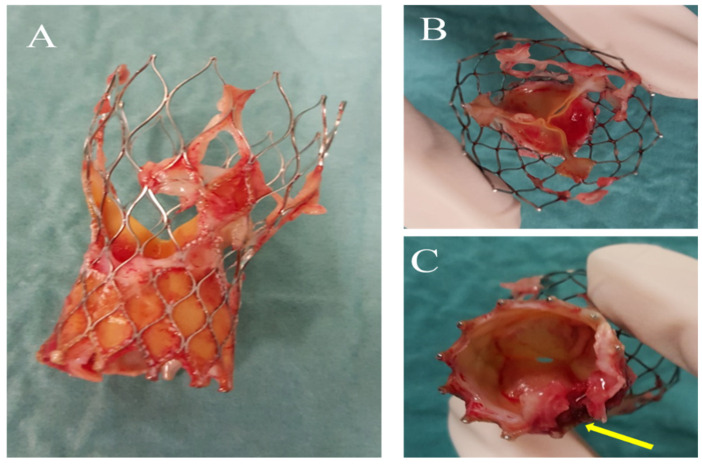
(**A**) TAVI prosthesis removed with evidence of vegetations attached on the frame. (**B**) Aortic view. (**C**) Ventricular view with evidence (yellow arrow) of valvular destruction in correspondence with the abscess in the intervalvular fibrosa.

During hospitalisation, the transthoracic echocardiogram (TTE) revealed no vegetations or para-valvular leaks. However, TAVI leaflet thickening and an augmented transvalvular gradient were observed. A CT brain scan showed two recent ischemic lesions, potentially linked to an embolic aetiology. On a transesophageal echocardiogram (TEE), a mobile vegetation was identified, attached to the frame of the transcatheter prosthetic valve, along with an abscess in the intervalvular fibrosa. Positive blood cultures for *Enterococcus faecalis* confirmed the diagnosis of IE. A fluorodeoxyglucose positron emission tomography/computed tomography (FDG-PET/CT) also confirmed multifocal uptake of the prosthetic. The patient was referred to a cardiac surgeon for consideration of aortic-valve replacement (Figure 1 and Figure 2). 

## 3. The Clinical Problem

*E. faecalis* is the most clinically significant pathogen among the enterococci, accounting for roughly 5–8% of hospital-related bacteremia cases and 5–20% of all endocarditis cases [15]. Endocarditis caused by *E. faecalis* results in an infection of the heart valves or inner lining, leading to valve damage and mortality if effective antibiotic treatment is not given. The emergence of resistance to multiple antibiotics has emphasised the significance of gaining novel perspectives into enterococcal endocarditis, a condition that poses a severe clinical threat. Therefore, it is crucial to explore alternate approaches to existing antibiotic strategies [8,9,25]. In this regard, feasible options may include obtaining therapies based on immunoprophylaxis or immunotherapy, which target proteins expressed in vivo and that are crucial for virulence.

Here, we address a case of a bacterial infection sustained by *Enterococcus faecalis*. We aim to expand understanding of bacterial endocarditis caused by Enterococcus species and review current evidence on the pathophysiology of *Enterococcus faecalis*-induced IE. Furthermore, we present an evidence-based algorithm to determine the optimal treatment approach.

## 4. Pathophysiology of *E. faecalis* Endocarditis 

The initial query is what classifies *E. faecalis* as a pathogen. In fact, the pathogenic action of *E. faecalis* is distinct in its execution. Unlike group A streptococci or Staphylococcus aureus which are highly virulent pathogens that rely on the secretion of numerous hemolysins and toxins to effectively counteract innate immune responses [26,27], *E. faecalis* employs a lesser number of virulence factors for pathogenesis [11]. Emerging evidence indicates that the initial stage of *E. faecalis* infection is primarily initiated by attaching and colonising the surfaces of host tissue [7,11].

Supporting this hypothesis, we detected predominantly gram-positive pathogens, indicating the important function played by proteins in the adhesive matrix molecules (MSCRAMM) family that may be potential targets for the development of new and effective immunotherapies [28]. Sillanpaa and colleagues [29] have identified 17 proteins from the *E. faecalis* V583 genome [12] possessing cell-wall-anchoring motifs and MSCRAMM-like structural features [12,29]. These proteins contain one or more segments of 150 to 500 amino acids, which have Ig-like folds that are typical of the MSCRAMM Ig family found in Staphylococcus aureus, as observed in studies [29,30]. The detection of antibodies in *E. faecalis* endocarditis patients’ sera that interact with some of these proteins provides a clear explanation that these antibodies are effectively expressed in vivo during the infection. The researchers observed that a large proportion of patients’ sera had particularly high titres against three of these proteins—EbpA, EbpB, and EbpC [29].

There is evidence to suggest that two protein adhesins, enterococcus surface protein (Esp) and collagen adhesion protein of *E. faecalis* (Ace), play a significant role in promoting the attachment of enterococcus to host tissues [31,32]. Additionally, the aggregation substance (AS) facilitates the aggregation of replicating bacteria [33], further complementing the work of these two proteins.

**Figure 3:**
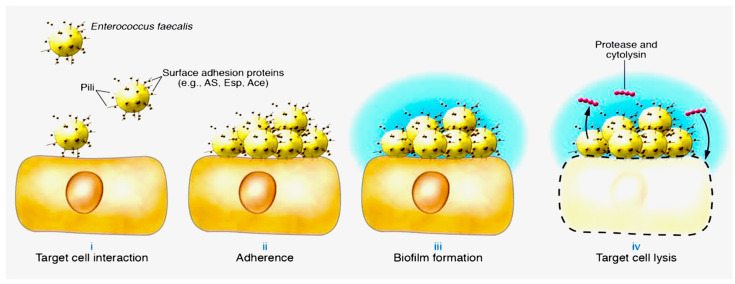
Role of pili in the establishment of *E. faecalis* infection is depicted with three pivotal actions. Enterococci may use pili acting as surface fibrils for microbial attachment to host tissues (**i**). Surface proteins (e.g., AS, Esp, and Ace) are involved in establishing tight bacterial adherence to host cells (**ii**). Enterococcal aggregation induces biofilm formation (**iii**) and elaboration of exopolysaccharide matrix (blue surface layer). Quorum-sensing-controlled expression of protease and cytolysin mediate host cell death and spread of infection (**iv**). Schematic modified with permission from ASM Press. From Budzik et al J Clin Invest. 2006 Oct;116(10):2582-4, Ref [31].

During enterococcal infections, the secretion of virulence factors that degrade tissue is of significant importance. Consistency in the presence of these factors is essential for optimal infection control. Protease expression regulation in Enterococci occurs through the quorum-sensing locus of regulators (fsr) in *E. faecalis*, which includes the *fsrC* sensory kinase, *fsrA* response regulator, and *fsrB*-derived autoinducer [12,34]. Carniol et al. [34] examined the pathogenic and biomolecular mechanisms following the creation of an infectious site that contains a certain amount of enterococci. The activation of protease secretion occurs through non-latent Fsr signalling. The subsequent stage is the development of a biofilm, facilitated by Esp, Ace, Fsr, and proteases. The authors noted that within the bacterial community formed and attached to the surface enclosed in an extracellular matrix, enterococci exhibit a slower growth rate, higher resistance to antibiotics, and a high frequency of lateral gene transfer [34]. Cytolysin is expressed during this phase, being a toxin secreted by enterococci, which is cytolytic in nature due to two peptides possessing modifications characteristic of lantibiotics. The latter is made up of thioether amino acids, which result from post-translational modification of precursors synthesised at the ribosomal level [35,36]. These peptides work together to break down several host cells, including polymorpho-nuclear leukocytes, which are a crucial part of the immune defense against *E. faecalis* infection (Figure 3) [14].

Microbiologists have recently confirmed the presence of pili on the surface of various strains of gram-positive bacteria, including *Streptococcus* spp., *Actinomyces* spp., and *Corynebacterium* spp. [37,38,39,40]. The presence of adhesins on the tip of the pilus, as well as the major and minor subunits of the pilus, has been proven by investigators using antibodies as detection reagents [41]. The analysis conducted on the genome has detected the structural genes that encode the pilina subunits in *C. diphtheriae* and *A. naeslundii*. These genes are situated in close proximity to the genes that code for sortase-like enzymes [41,42,43].

Sortases function as transpeptidases in the cell wall envelope of gram-positive bacteria, like staphylococci, listeria, or bacilli. Their main role is to cleave selection signals of surface proteins, thereby blocking their C-terminal carboxyl group through amide bond formation [44,45]. Deleting the sortase or pilin subunit gene expression removes pili formation [41]. Ton That et al. [41] reported a common pilin motif sequence with a preserved lysine residue in all major pilin subunit genes, which is essential for pili formation. Additionally, Ton That et al. [46] demonstrated that gram-positive pili are resistant to SDS or organic solvents and can only be cleaved by proteases through biochemical tests. Together, these data suggest the development of a sortase-linked model, as emphasised in a subsequent report by Ton That and colleagues [47].

The importance of sortases in facilitating pilus assembly has been highlighted by their ability to generate transpeptide bonds between cleaved pilin precursors’ C-terminal carboxyl group and the amino group of lysine residues’ side chain in pilina motif sequences. This model allows for several predictions that significantly back the more aggressive nature of pathogens capable of processing it. Gram-positive pathogens are mainly involved, with their genomic sequences possessing gene clusters encoding sortase, as well as surface proteins with sorting signals and pilin motif sequences that produce pili [47]. This forecast has been supported by various studies focusing on the crucial role of pili in promoting the pathogenicity of enterococci [48], and identifying pili’s presence on the surface of group B streptococci or pneumococci [49,50].

### 4.1. Enterococcal Pili and Infective Endocarditis

Sillanpää and colleagues [29] identified three open reading frames, ef1091–ef1093, that showed sorting signals and pilin motif sequences. Patients infected with *E. faecalis* were found to have a higher frequency of antibodies against these proteins in their sera compared to uninfected control patients [29]. Nallapareddy and colleagues [48] advanced the research on f1091–ef1093 and Sortase C (srtC), demonstrating that these three open reading frames and srtC are constituents of an operon that encodes pili associated with endocarditis and biofilm formation (ebp). Using immunofluorescence electron microscopy, researchers found EbpA, EbpB, and EbpC pili present on the surface of *E. faecalis*. Biofilm assays revealed that mutants lacking pili were unable to form biofilms. A rat endovascular infection model was developed to study the mechanisms of IE determination, with microbes recovered from aortic vegetations. Pilus mutants were found to have reduced competitiveness compared to wild-type enterococci, indicating the necessity of pili for enterococcal-infection establishment. A study using specific probes detected ebpA, ebpB, and ebpC genes in 408 *E. faecalis* isolates from four continents, leading to the conclusion that pili formation may be a widespread trait of *E. faecalis*.

Similarly, Tendolkar and colleagues [51] recognised a cluster of surface protein and sortase genes implicated in the formation of biofilms, which also contained the biofilm enhancer in Enterococcus. Nevertheless, this study did not demonstrate the capacity of enterococci to create pili [51]. Recent research supports this hypothesis and shows that immunising animals with the pilin subunit can prevent both neonatal infectious meningitis caused by group B streptococcus and infection caused by group A streptococcus [52,53]. The discovery of pili and pili assembly in gram-positive bacteria offers the promise of new technologies and may also trigger promising public-health interventions for humans.

Several studies have indicated that the ability of *E. faecalis* strains to form biofilms is a critical factor in promoting infection, in addition to the MSCRAMM-mediated colonisation process [34,54,55]. Mohamed et al. [55] examined biofilm formation in a large number of *E. faecalis* isolates and found that endocarditis isolates produced biofilms significantly more frequently and to a greater extent than non-endocarditis isolates. Seven genes or gene clusters linked to *E. faecalis* biofilm formation have been confirmed by multiple studies [51,55,56,57,58,59].

### 4.2. Lesion Development and Progression of Infective Endocarditis in Heart Structure

Enterococci act as causative pathogens, causing infectious foci with aggressive colonisation due to their unique biogenesis. Standardisation of language and adherence to specific units and metrics are crucial in understanding the severity of the ailments caused by these bacteria. *Enterococcus faecalis* is the most prominent strain, resulting in both native valvular endocarditis (NVE) and prosthetic valvular endocarditis (PVE) in elderly or chronically ill patients [1,2,3,5]. The lesions commonly exhibit progressive evolution, forming large abscess cavities that affect one or more valves. In the most aggressive forms of IE, extensive parts of the heart, such as the aortic root, the intervalvular fibrosa, and the heart trigones, are destroyed [60,61,62,63,64,65]. It is well established that the evolution of *Enterococcus faecalis* infection is caused by the increasing resistance that these pathogens often develop towards vancomycin, aminoglycosides, and ampicillin. This has been documented in numerous studies [8,9,12,51,54,55,56,57,58,59].

The normally resilient cardiac endothelium is susceptible to bacteremia during *E. faecalis*-induced IE associated with an underlying colon tumor. Following endothelial injury, inflammatory cytokines and tissue factors are released, resulting in fibronectin expression and the formation of a platelet-fibrin thrombus that promotes bacterial adhesion [37,39,66]. Endothelial damage occurs due to the presence of valvular sclerosis, rheumatic valvulitis, or direct bacterial activity, commonly in cases of IE caused by *Staphylococcus aureus*, strains of *enterococcus* spp. (such as *E. faecalis*, Group D streptococci *S. gallolyticus*, *S. bovis*), or *Streptococcus viridans* (including *S. mutans*, *S. salivarius*, *S. anginosus*, *S. mitis*, and *S. sanguinis*). Bacterial colonisation leads to more rounds of endothelial damage and clotting, ultimately resulting in infected growth [37,39,66].

**Figure 4:**
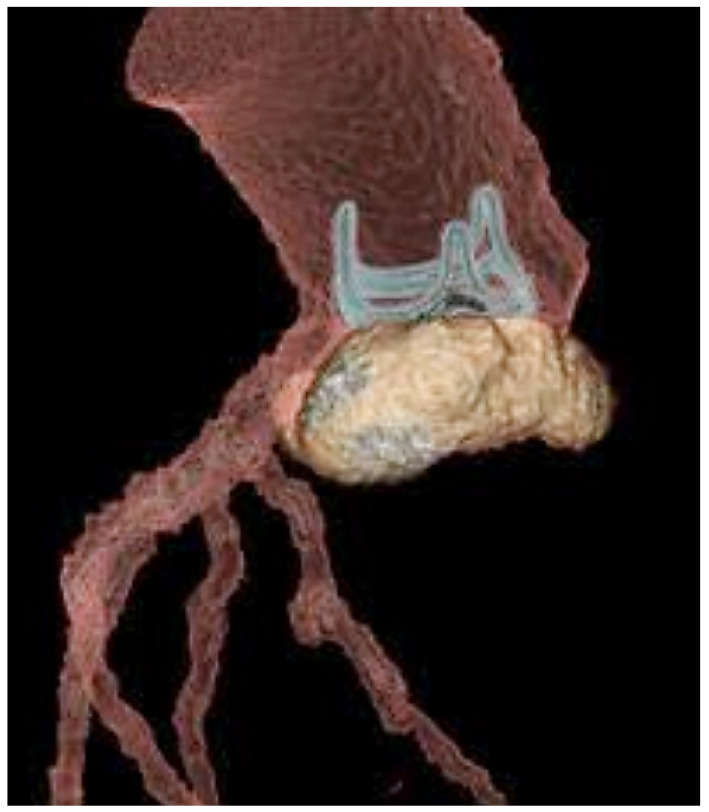
Patient with PVE due to *E. faecalis*. Biological aortic-valve prosthesis with posterior semilunar abscess.

**Figure 5:**
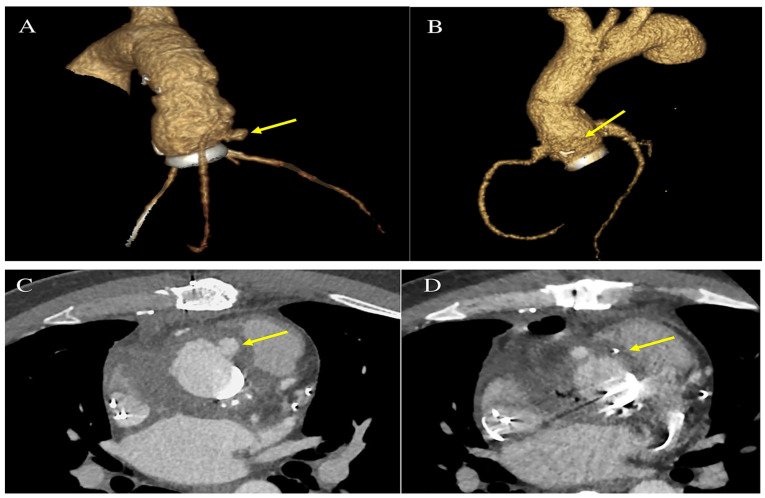
PVE from *E. faecalis*. (**A**,**C**) Three-dimensional CT shows a pseudoaneurysm (yellow arrow) before surgery. (**B**,**D**) Three-dimensional CT after surgery with complete removal of the pseudoaneurysm (yellow arrow) and replacement of the mechanical prosthesis. Abbreviations: CT, computed tomography; PVE, prosthetic-valve endocarditis.

The formation of a biofilm, consisting of layers of bacteria held in a matrix of polysaccharides and proteins, can aid in bacterial survival and increase resistance to antibiotics. This suggests that pili attached to sortase, previously discovered in *Corynebacterium* spp., *Actinomyces* spp., group A and group B streptococci, and pneumococci, may also contribute significantly to the development of enterococcal infections [67]. Nallapareddy et al. [48] found that *E. faecalis* pili have a crucial role in biofilm formation and mediating endovascular infection, facilitating successful colonisation of the valve endothelium. If *Enterococcus faecalis* infection spreads beyond the valve annulus, it could result in an abscess, pseudoaneurysm, fistula, or atrioventricular block. The formation of a pseudoaneurysm may occur as a perivalvular cavity connected to the cardiovascular lumen, detected via colour Doppler flow observed during echocardiography, or as an abscess with no such communication with the cavity thickened and filled with pus. In Figure 4 and Figure 5, we illustrate an example of IE induced by *E. faecalis* [62,63,64,65].

In severe cases, the formation of a fistula can cause a critical progressive infection around the heart valve (known as aorto-cavitary fistula) and has a mortality rate exceeding 40%, even with surgical intervention [68,69,70,71]. The identification of *E. faecalis* pili and sortase-anchored pili is of utmost importance as these surface proteins, found ubiquitously, are antigenic in humans during endocarditis infection [48]. Thus, the potential for new preventive measures to impede the pathogenesis of enterococcal endocarditis offers opportunities for future prevention and treatment strategies.

## 5. Clinical Evidence: Imaging Criteria

Infective endocarditis is a complex condition with notable morbidity and a high mortality rate during hospitalisation. Management of IE requires a multidisciplinary approach, a dedicated Endocarditis Team, and prompt access to advanced imaging techniques. Early surgical intervention when deemed necessary can be beneficial in reducing in-hospital mortality [24,71]. Patients with infective endocarditis (IE) caused by *E. faecalis* should undergo a detailed assessment of symptoms and a transthoracic echocardiogram (TTE). This will primarily evaluate the development of vegetations affecting one or more leaflets, the extent of the infection in the heart and aorta components (such as the leaflet, annulus, trigones, intervalvular fibrous, left atrium, and aortic root), as well as the size and function of the left ventricle.

Echocardiography remains the primary imaging technique for detecting anatomical evidence of infective endocarditis [19,68,69,70]. It is also a crucial Major Criterion in the 2023 Duke-ISCVID IE Criteria. While valvular vegetation is the typical echocardiographic sign of infective endocarditis (IE), other complications affecting valvular leaflets (such as perforation or pseudoaneurysm), paravalvular structures (such as abscess or fistula), or prosthetic valves (like valvular dehiscence) can also signal IE [5,69]. Transthoracic echocardiography is less sensitive than transesophageal echocardiography (TEE) for diagnosing IE.

Therefore, transesophageal echocardiography (TEE) is generally necessary when infective endocarditis (IE) is suspected, particularly in cases involving prosthetic valves, cardiac devices, or suspected complications such as perforation, paravalvular lesions, fistula, and prosthetic-valve dehiscence [5,19,68,69,70,71]. Recent studies have demonstrated an IE prevalence rate of up to 33% in many patients with hematogenous spondylodiscitis; therefore, TEE is also recommended in these cases [69]. Although TEE is highly sensitive and specific, complex clinical scenarios may arise where echocardiography cannot confirm or rule out the diagnosis of IE. In such circumstances, as well as for all instances of IE in patients with intracardiac implants or suspected paravalvular extension, additional diagnostic approaches may be useful in affirming the diagnosis [71].

TEE assesses abscess development and progression, and the mechanism and severity of valve regurgitation. It is noteworthy that TTE shows moderate sensitivity (75%) and specificity (over 90%) in the detection of vegetation that confirms suspected native valvular endocarditis, as observed in studies [68,70,71,72]. Patients who present negative or equivocal evidence of infection on TTE, yet possess a high clinical probability of IE, should undergo TEE given its sensitivity of more than 90%. A negative TEE indicating the absence of vegetations is a robust predictor of the absence of disease; however, if clinical suspicion is high, repetition of the examination 7–10 days later is necessary to confirm diagnostic negativity. If this test is still negative, it is possible to rule out the diagnosis of IE. Further echocardiography would not provide any additional useful information. As the specificity of TEE does not reach 100%, excluding false positives is necessary for a differential diagnosis [5,68,70,71,72,73,74,75,76].

**Figure 6:**
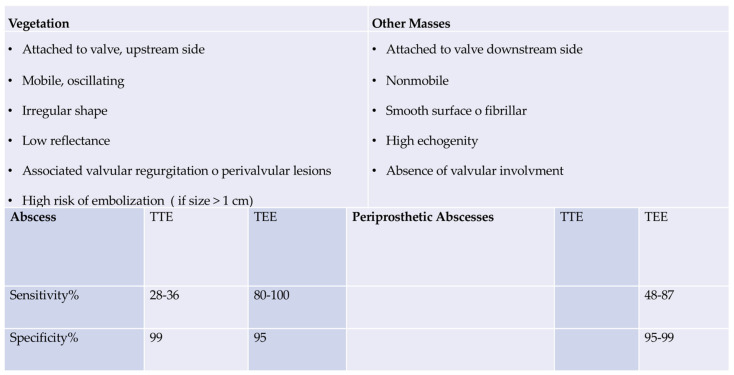
Sensitivity and Specificity of Echocardiography in detecting abscesses. Fowler, V.G. et al *Clin. Infect. Dis.* 2023, *77*, 518–526. Ref [5].

For the assessment of developing lesions, TEE is superior to TTE in detecting significant cardiac issues such as abscess, leaflet perforation, and pseudo-aneurysm. As a result, in most cases, TEE should be performed even if TTE has already provided enough indications for a definitive diagnosis. Research suggests that in the case of suspected endocarditis in patients with prosthetic valves, the TTE’s sensitivity is inadequate, not exceeding 36–69%. Therefore, a TEE is frequently required. [68,70,71,72,73,74,75] The selection of TEE is crucial in the case of a heart-device infection [5,68,71,72,73,74]. Moreover, TTE must be repeated if a complication is suspected, and after the completion of therapy as a baseline for follow-up. Please refer to Figure 6 Ref. [5]. 

Today, it is strongly recommended to complete the diagnostic procedure with a CT scan, an 18F-FDG-PET/CT, and a cardiac MRI. The ISCVID Working Group included cardiac computed tomography (CCT) as a supplementary imaging modality in the 2023 Duke-ISCVID IE Criteria. Although CCT’s capability to detect vegetations is inferior to that of echocardiography, it exhibits a greater sensitivity in detecting paravalvular lesions due to its enhanced spatial resolution [68,75,76,77]. For instance, CCT was more effective in diagnosing pseudoaneurysm or abscess than TEE, with a sensitivity of 78% vs. 69%. However, TEE was superior to CCT in detecting vegetations (94% vs. 64%), valvular perforation (81% vs. 41%), and paravalvular leakage (69% vs. 44%). The combination of computed tomography and echocardiography has been found to be more sensitive than either modality alone in diagnosing all valvular and paravalvular lesions [5,68,71]. Thus, the ISCVID Working Group views these two imaging modalities as complimentary for suspected infective endocarditis cases. Moreover, CCT may serve as a helpful supplement in situations where TEE is contraindicated or when TEE images are suboptimal due to calcifications or intracardiac implants.

**Figure 7:**
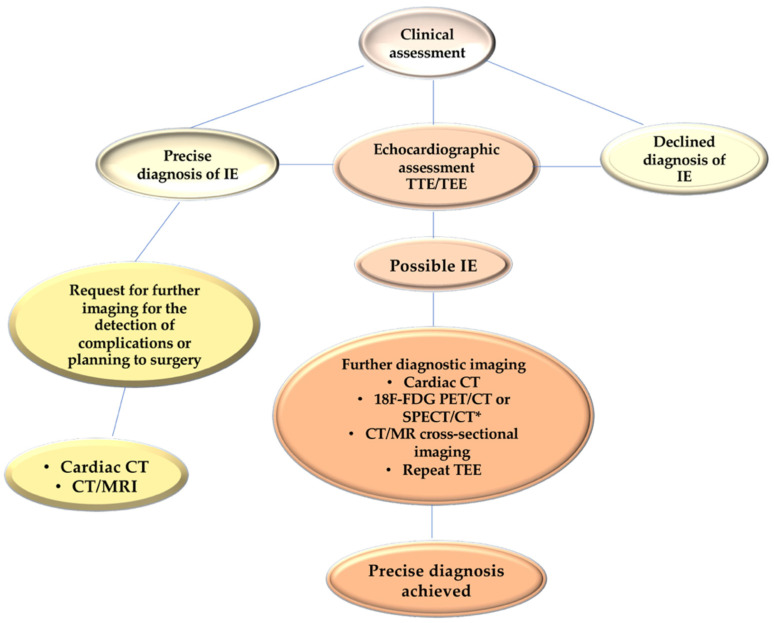
The applied strategy of integrated imaging in patients with suspected infective endocarditis IE. In patients included in the subgroup with possible IE after initial evaluation by TTE and TEE, cardiac CT imaging, metabolic imaging, or transverse imaging of the head and viscera by CT scan or MRI is indicated to achieve a precise early diagnosis. For suspected IE, 18 FDG-PET/CT or cross-sectional imaging via CT or MRI (or metabolic imaging) scans may assist with the detection of complications, such as abscess, mycotic aneurysm, infarct, or hemorrhage in patients with definite IE. Abbreviations: IE, infective endocarditis; FDG-PET/CT, fluorodeoxyglucose positron emission tomography/computed tomography; MRI, magnetic resonance imaging; TEE, transesophageal echocardiography; TTE, transthoracic echocardiography. From Nappi et al. Ref [62,63,64,65,74,75]. * Delgado et al Eur Heart J. 2023 Aug 25, Ref. [68].

Positron emission CT with 18F-fluorodeoxyglucose ([18F] FDG PET/CT) has been incorporated as an imaging modality in the 2023 Duke-ISCVID IE Criteria. [18F] FDG PET/CT surpasses the diagnostic shortcomings of echocardiography when prosthetic material is being evaluated [37], thus resulting in the reclassification of a substantial section of suspected PVE cases from “possible” to “definite” IE. Due to ongoing controversies surrounding the efficacy of [18F] FDG PET/CT in rejecting IE, the ISCVID Working Group is currently prioritising investigations into its positive predictive value. The addition of [18F] FDG PET/CT as a Major Criterion in the Duke Criteria is shown to have a significant improvement in identifying definite PVE (pooled sensitivity, 0.86 [0.81–0.89]; pooled specificity, 0.84 [0.79–0.88]) when compared to echocardiography alone [5,68,78]. [18F] FDG PET/CT holds particular significance in diagnosing cardiac infections in patients with intricate cardiac implants, like multiple prosthetic valves, combined aortic valves and grafts, and congenital heart disease [5,68,78,79,80,81] (Figure 7).

## 6. Clinical Use: Treatment Option at referral Centre for IE

Surgery is necessary for 40–50% of patients with IE [82]. Prior to the introduction of valve-repair procedures, valve replacement was the favoured option for severe valve regurgitation due to the likelihood of IE recurrence [71,83]. Valve replacement may be preferred in specific circumstances, including cases of advanced age, or when a combined or complex surgical procedure is required, involving complete tissue removal in cases of PVE. It may also be recommended in instances of extensive and harmful NVE when infectious fields encompass a significant portion of the damaged heart [24,71,82,84,85,86,87,88,89,90]. The latest treatments employ transcatheter-valve therapy (TVT) to treat structural heart disease, which has demonstrated itself as a secure and useful technique for many patients. However, it is preferable to limit its use to elderly patients with coexisting medical conditions. Patients are referred to centres specialised in the use of TVT because this group of individuals is potentially exposed to a high risk of mortality and complications after undergoing the standard surgical approach [91].

**Figure 8:**
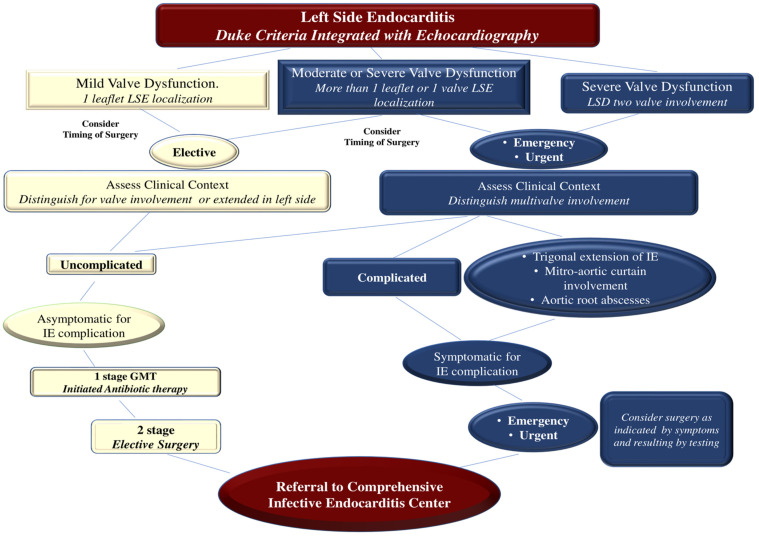
Take-Home Messages and Clinical Algorithm for the Management of Left-Side Endocarditis due to GPC. Abbreviations: GMT, guide medical therapy; GPC, gram-positive cocci; LSE, left-side endocarditis. Other abbreviations are listed in other figures. From Nappi et al. Refs. [62,63,64,65,71,78,79,80,81].

The accomplishment of valve surgery for IE is based on four general principles. First, surgical procedures must ensure the complete removal of infectious vegetations, followed by the repair or replacement of one or more heart valves. Secondly, the full integrity of the cardiac structures should be restored. Thirdly, to prevent the relapse of infection, complete debridement of the infected tissue should be performed. Valve replacement with allogeneic or autologous tissue is recommended to restore full cardiac function. Finally, if a surgeon performs a valve repair, they must ensure that there is no more than a trace of mild valve regurgitation after completing the repair [65,71,83,84,85,86,87,88,89,90] **(**Figure 8).

### 6.1. Shared Decision-Making in IE Caused by Enterococcus faecalis

*E. faecalis*-induced endocarditis often presents a severe clinical picture that requires urgent hospital admission to a referral centre for endocarditis treatment. Delaying surgical procedures is not advised, and patients with IE from *E. faecalis* may experience heart failure due to regurgitation or valve obstruction, which is the main indication for surgery. Reported evidence from historical cohorts has demonstrated that the consequence of delaying surgery beyond 24 h of hospital admission could be catastrophic. These patients could develop refractory pulmonary edema or cardiogenic shock, leading to a rapid deterioration in their clinical status [92,93]. Patients with well-tolerated, severe valvular regurgitation resulting from extensive *E. faecalis* vegetations might be suitable for postponed surgery following a period of stabilisation with antibiotic therapy. However, recent evidence suggests surgical treatment should happen within 48 h [71,89,94,95].

The prevention of embolism is the second concern of *E. faecalis* IE driving surgery. This complication affects 25–50% of patients and can result in infarction of the end organs such as limbs, spleen, kidney, and coronary arteries. Stroke is the most frequent neurological complication, but embolic complications can arise in any vascular bed [71,89,96]. Moreover, vegetation embolism could result in the development of a mycotic aneurysm as a long-term complication of the localised vascular-wall infection. These aneurysms are typically found in cerebral vessels and are detectable through brain-imaging techniques in 3–5% of individuals with IE caused by *E. faecalis*, with the majority remaining asymptomatic [97,98,99,100,101].

The onset of *E. faecalis* endocarditis on the right side of the heart could considerably increase the likelihood of emboli to the lungs or systemic circulation via a patent foramen ovale. The majority of emboli are observed within the first 2 weeks of diagnosis and the risk quickly diminishes once antibiotics treatment is initiated [100,101]. In cases of embolism resulting from IE caused by *Staphylococcus aureus* or *E. faecalis*, the vegetations that contribute to the embolic process are typically large (over 10 mm in length), highly mobile, and commonly found on the mitral valve [71,102]. Surgery may be necessary if emboli continue to occur, and patients display evidence of persistent threatening vegetations as shown by echocardiography. Surgery is now considered safe after an ischemic stroke and the recommended delay of at least one month to avoid cerebral hemorrhage has been reassessed and significantly reduced intervention times are now in place [71,89].

Managing *E. faecalis*-induced IE is particularly challenging after TAVI, given the complexity of diagnosis and decision-making in this patient group. Therefore, the considerations for managing this specific type of IE are even more vital. TAVI-IE is a newly emerging entity, which is distinctly different from standard aortic-valve surgery–IE in terms of clinical features, microbiology, risk factors, and outcomes [68,103]. Higher exposure to healthcare procedures, older age, higher rates of comorbidities, and technical factors linked to the procedure can lead to an increased risk of bacteremia, which may contribute to increased risk of TAVI-IE [68,104,105,106,107].

Current guidelines do not provide any specific recommendations for this condition, which poses challenges in terms of diagnosis and management. The modified Duke criteria are less useful for diagnosing TAVI-IE due to reduced sensitivity of TTE and TEE caused by the metallic frame artifacts [5].

While early surgical intervention is advisable, most patients are treated with intravenous antibiotics alone, owing to advanced age and high surgical risk [105,106]. This may partly account for the elevated rate of mortality in hospital (36% to 63.6%). These findings indicate that a more assertive strategy and prompt diagnosis in these patients are necessary to enhance clinical results.

The exact role and timing of surgery are still debated. Currently, surgical intervention is only recommended after meticulous individual case analysis, and it is unclear which patients will benefit from surgery. TAVI-IE is also a technical challenge, mainly due to the stent frame’s adherence to the aorta (particularly for self-expandable TAVI) and mitral-aortic fibrosa removal, particularly in inflamed tissues. There is a consensus that there is no disparity in TAVI-IE incidence between the two types of TAVI prosthesis. Nonetheless, two studies indicate a greater prevalence of TAVI-IE and vegetations attached to the stent frame among patients who receive self-expandable prostheses [105,106]. Although we cannot provide a clear explanation for these findings, we concur that the larger stent frame found in self-expandable devices, coupled with the larger contact surface between the frame and native tissues, may act as potential anchoring and disseminating points during bacteremia [105,106,107]. It is believed that there is no difference in the occurrence of peri-annular complications between the two types of valves. However, in the case of self-expandable prosthesis implantation, there is a higher risk of an aortic tear in the presence of inflammatory tissues.

### 6.2. The Use of Biological Substitutes in the Context of E. faecalis Infective Endocarditis

Surgery can be very challenging in cases of abscesses that develop in the aortic annulus due to the colonisation of *E. faecalis*. This colonisation often leads to detachment of the intervalvular fibrosa, involvement of the aortic root and fibrous trigones, as well as the formation of a left atrial fistula. The surface pili of *E. faecalis* attract mating cells, promoting bacterial union, and leading to the formation of abscess cavities which may invade large areas of the heart [35,39,48]. Furthermore, the growth and expansion of pili-dependent infectious fields in *E. faecalis*-induced IE results in lesions larger than 10 mm, affecting multiple valve leaflets and often causing a severe inflammatory response [3,10,11,48]. This inflammatory response also affects the perivalvular tissues, causing them to become extremely fragile. The annulus’ consistency, as well as that of the surrounding regions, cannot be relied upon for suturing [63,71,72,84,108,109]. The anatomical-pathological features of *E. faecalis*-induced IE are observed in both NVE and PVE, with prosthetic-valve endocarditis affecting 3–4% of patients within 5 years of index surgery. The condition can affect mechanical and bioprostheses similarly. More than a third of cases are acquired in the healthcare setting [68,79,80].

Recently, there has been an increase in early *E. faecalis*-induced IE occurring in prosthetic valves with a latency of less than one year after initial surgery. Previous reports have emphasised the dominant role played by coagulase-negative *S. Aureus* colonisation during the first 2 months after surgery [110]. However, this trend has been challenged recently in cases of endocarditis- and biofilm-associated pili infection caused by *Enterococcus faecalis* [48].

Over the course of one year, the range of pathogens causing PVE is equivalent to that of NVE, with *enterococcal* spp. being predominantly present [5]. As indicated in the case report, the clinical presentation is frequently upheld by extraordinary and negative imaging outcomes, in addition to the not-so-evident Duke criteria [5,111,112]. The formation of root abscesses and progression to valve dehiscence is common, with up to 60% of patients experiencing this occurrence rate. Surgery is typically necessary and performed before the patient’s clinical condition declines within 48 h of diagnosis. The surgical procedure is frequently technically challenging and high-risk, and recurrent PVE rates can vary between 6% and 15% [82]. PVE mortality is exceedingly high, especially when it is caused by S aureus, in comparison to *enterococcal* spp. One-year mortality rates can reach 50% [84,108,113,114].

To prevent infection recurrence caused by *S. aureus* and *E. faecalis* colonies, a biological substitute such as an aortic homograft or another full root xenograft is recommended for these patients. However, the clinical effectiveness of using an aortic homograft in the field of IE remains uncertain due to the absence of RCTs [103,108,115,116,117,118,119,120,121]. A recent report from the Endocarditis Study Group at the Cleveland Clinic suggests that the use of allogeneic tissue can reduce the risk of infection relapse [122]. The STS database report between 2005 and 2011 shows a decreasing use of homograft in first-time aortic-valve replacement for IE (from 9.4% to 5.6%) and in reoperation (from 37.5% to 28.5%). Nevertheless, patients who require reoperations are more likely to receive an aortic homograft than those undergoing primary interventions (32.2% vs. 7.0%, *p* < 0.0001), for both valve replacements (14.6%) and root replacements (53.2%).

It is worth noting that no significant disparities in overall mortality and recurrence of infection have been reported when comparing conventional mechanical and biological substitutes in IE [108,115,116]. Klieverik and his team [119] found that patients who received homografts had a similar rate of recurrent endocarditis compared to those with mechanical valves, but with a lower freedom from reoperation (76% vs. 93%, respectively). Sabik and colleagues [120] reported on a study of 103 patients with prosthetic IE, almost 78% of whom suffered from periannular and root abscesses.

These patients were managed with aortic homografts and experienced a freedom from recurrent infection rate of 95% at over two years, and an operative mortality rate of 3.9%. Fukushima et al. [117] found reinfection rates of just 0.2% at 30 days, but 5.5% of patients suffered from late infections at a median time of 5 years (ranging from 4 months to 16 years) after allograft implantation. Arabkhani and colleagues [118] reported favourable outcomes 27 years postoperatively utilising aortic homografts, with a low reoperation rate for recurring infections (2.2%). Allogeneic tissues demonstrated favourable responses to antibiotic treatment, effective in 21–25% of cases [103,108].

Musci and colleagues [123] and Yanka and colleagues [124] were the first to report systematic utilisation of homograft aortic-root replacement for the treatment of active endocarditis and peri-annular abscess. In their study, Musci and colleagues employed allografts in 221 out of 1163 patients, resulting in a lower rate of infection recurrence (5.4%) in both native-valve endocarditis (NVE) and prosthetic-valve endocarditis (PVE), with a 10-year freedom from reoperation rate of 92.9% ± 3.2% and 92.1% ± 2.5%, respectively. Early mortality was 16% for NVE and 25% for PVE, but 10-year survival was better in NVE compared to PVE (47% vs. 35%). Notably, over 25% of deaths occurred during intraoperative, indicating the complexity of this surgery among critically ill patients. Additionally, Yanha and colleagues [124] demonstrated exceptional clinical performance and durability with a low reinfection rate and late mortality of 7.9%. Patients had a survival rate of 97% at 1 year and 91% at 10 years, respectively.

There are few contraindications for allograft use in IE patients. The allogenic substitute has low reinfection rates within one year and good hemodynamic performance [114,116]. The peri-annular abscess with intervalvular fibrosa involvement in PVE is the most preferred target for allogenic-tissue implantation, but it can also be used in NVE scenarios [84,109,114,116,120,125,126,127].

We have outlined the usage of cryopreserved aortic or mitral homografts to replace diseased valves in 56.2% and 21% of patients with abscess formation. Additionally, in cases of aggressive IE with extension to the aorto-mitral junction and mitral valve, a double homograft was utilised [63,64,71,109,114,125,126]. Among the recipients, two thirds received monobloc implants [109,114,125,126], while the remainder had separate blocs inserted with partial mitral homografts. The implant technique yielded favourable outcomes, even when dealing with delicate tissue.

**Figure 9:**
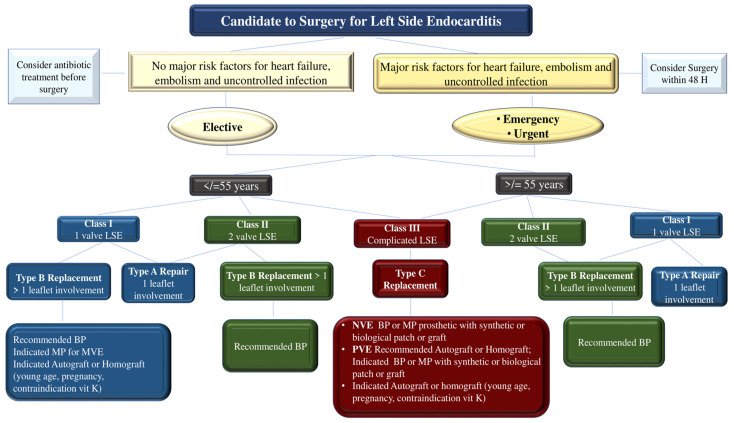
Take-home messages and clinical algorithm for the choice of optimal valve substitute for left-side endocarditis due to GPC. Abbreviations: BP, bioprosthetic; GPC, gram-positive cocci; MP, mechanical prosthetic; NVE, native-valve endocarditis; PVE, prosthetic-valve endocarditis. Other abbreviations are listed in other figures. From Nappi et al. Refs. [62,63,64,65,71,78,79,80,81,109,114,125,126].

The use of allogeneic tissues in severe heart infections, whether in natural or artificial valves, is endorsed by Steffen et al. [128]. Post-implantation, the allogenic tissue exhibited a notable anti-bacterial effect, even after a storage period of five years. Antibiotic combinations administered during allograft treatment suggest a substantial impact on their resistance to infection. Tests conducted on ascending aortic homograft tissue have shown a significant increase in resistance to staphylococcal and enterococcal bacteria (*Enterococcus faecalis* and *S. aureus*), with lower bacterial contamination compared to homograft aortic valves. Kuhen and colleagues [129] proposed that administering antibiotics after thawing an allograft can significantly reduce infection recurrence, a benefit not yet demonstrated with conventional prostheses or Dacron grafts. However, using antibiotics to pretreat the prosthesis can reduce the risk of vascular-graft infection. The mechanism for this beneficial action and its potential interaction with pili function in *E. faecalis*-induced IE are unknown (Figure 9).

## 7. Discussion

*E. faecalis*, a common nosocomial pathogen associated with the development of bacterial endocarditis, highlights the necessity for alternative therapeutic approaches, like immunoprophylaxis or immunotherapy. While empirical antibiotic therapy should be initiated immediately upon blood-culture acquisition, clinicians may opt for culture-guided treatment if the patient is clinically stable [16]. Although empiric antibiotic regimens for native-valve endocarditis and prosthetic-valve endocarditis are based on definitive guidelines [16] published by the British Society for Antimicrobial Chemotherapy, the demonstrated resistance of *E. faecalis* to antibiotic treatment suggests the need to acquire new knowledge on the biogenesis and resistance of *E. faecalis* for future directions.

It is highly recommended to include molecular biology knowledge in conjunction with microbiology in the shared decision-making process alongside microbiological specialists. Intravenous combination therapy is generally preferred over monotherapy to minimise resistance and offer antimicrobial synergy [130]. Currently, there is encouraging laboratory data but limited clinical evidence to support the use of combination beta-lactam therapy for this indication. Further research is needed to determine the potential benefits of combining beta-lactam therapy contrasted with monotherapy to treat Gram-positive blood infections. Nonetheless, in cases of bacteremia unresponsive to standard antibiotic treatment, combining therapy may be advantageous [130]. The only exceptions are *S. aureus* and *E. faecalis*, as they are vulnerable to methicillin. Other treatment options for infections that have become resistant to vancomycin are obtainable, including linezolid, tigecycline, and daptomycin [131,132].

Although beta-lactamase resistance is rare in *Enterococcus faecalis* infections, a recent study used RT-PCR to detect antibiotic resistance genes (CTX-M, Van A, and Van B) in *Enterococcus faecalis* obtained from children with bacteremia. This is in contrast to pathogenic *Escherichia coli* ST131, which actively secretes CTX-M-15 β-lactamase [133]. A study by Sulainam et al. [133] revealed that of the *E. faecalis* isolates tested, 91.67% were susceptible to levofloxacin, 83.33% to amoxiclav, 66.67% to erythromycin, 58.33% to amikacin, 50% to ampicillin, and 33.33% to cefotaxime and ceftriaxone, respectively, while only 25% were susceptible to vancomycin. The study found that 88.89% of the nine vancomycin-resistant isolates were associated with the Van A gene, identified via real-time PCR analysis (*p* < 0.001). Notably, two important points should be considered. Firstly, 77.78% displayed Van B gene production, as identified via real-time PCR (*p* < 0.001). Secondly, all *E. faecalis* isolates resistant to cefotaxime and ceftriaxone produced the CTX gene, as detected via real-time PCR (*p* < 0.001) [133]. Recent studies demonstrate that a significant proportion of antibiotic-resistant genes in bacteria can be attributed to genetics. The transfer of genetic material between bacteria, via transformation and transduction, is considered the main factor responsible for antibiotic resistance in the majority of bacterial strains [134,135,136].

Given the rise in antibiotic resistance, there has been a growing interest in microbiological research that focuses on using bacterial factors as immunotherapeutic targets. This decision is based on the fact that bacterial factors play a significant role in an organism’s ability to colonise, infect, and ultimately cause disease [28]. MSCRAMMs have received significant attention recently due to their widespread presence and unique ability to promote the initiation of infections, including endocarditis [33], in both traditional and opportunistic pathogens [28,137]. Their central role in these processes is of particular interest. Unfortunately, complications have been identified in isolating and defining MSCRAMM from *E. faecalis*, which has yielded limited success due to this microorganism’s lack of adherence to ECM proteins in laboratory growth conditions [34,135]. This stands in contrast to its relatives, such as staphylococci and streptococci, which exhibit enhanced aggression.

To overcome this challenge, Sillanpa and colleagues utilised a bioinformatics method to identify multiple proteins that predict MSCRAMM-like structures [29]. By evaluating their reactivity with sera from *E. faecalis*-infected patients, the researchers concluded that some of these predicted proteins are indeed expressed by *E. faecalis* during infection. In particular, the study conducted by Sillanpa and colleagues [29], which examined antibodies in the sera of patients with *E. faecalis* endocarditis, identified nine recombinant forms of proteins anchored to the cell wall of *E. faecalis*. The authors noted three genes and a Sortase C (SrtC) gene, associated with sortase, which were expressed in vivo. Therefore, by showing the presence of antibodies against three of these proteins, with considerably elevated levels in the majority of infected patients’ sera [29], the authors have opened up avenues for further research.

Research has shown that *Enterococcus faecalis* virulence is enhanced by cell-wall-linked proteins, such as sortase-mediated endocarditis and biofilm-linked pilus (Ebp), which play a crucial role in biofilm formation both in vitro and in vivo. Furthermore, a substantial body of contemporary literature has reported a rise in multi-drug resistance in the fight against *Enterococcus faecalis* infections. The creation of biofilms is a particular concern because it not only has the potential to protect drug-resistant organisms from antibiotics and opsonophagocytosis, but it can also increase horizontal gene transfer [34,138]. Previous studies on *E. faecalis* have demonstrated that various factors can significantly reduce the density of biofilms [55,56,57,58,59]. Disruption of esp, which encodes the surface protein of Enterococci in certain strains, promotes the latter. This promotion is facilitated by various factors including the fsr 2-component system, gelE which encodes for gelatinase, the *Epa* gene cluster which encodes for the polysaccharide Epa, and finally by atn which encodes for an autolysin or bopD sugar-binding transcriptional regulator [55,56,57,58,59]. A recent study identified traits related to high biofilm production in the strain E99 through transposon mutagenesis. The study highlighted a gene cluster called bee, which had a similar organisation to Ebp [51]. However, this gene cluster was found to be uncommon in *E. faecalis* and instead present in a conjugative plasmid. Studies associating genes with clinical isolates show that biofilm formation in *E. faecalis* does not require either esp [55] or fsr/gelE [139]. This variation, noted in the presence or absence of numerous genes related to biofilm, may explain the species’ varying ability to form biofilm to some extent.

Recently, Nallapareddy et al. [48] conducted a study on mutation and complementation analysis, which illustrated the significance of the ebp operon, encoding endocarditis- and biofilm-associated pili, as well as srtC for biofilm formation in the *E. faecalis* strain OG1RF. Additionally, using immunogold electron microscopy with antisera against EbpA-EbpC proteins and patient serum, *E. faecalis* was found to generate pleomorphic superficial pili. It is worth noting that the Ebp protein cross-linking mechanism of SrtC favours the assembly of pili and their attachment to the cell wall. Significantly, a non-piliated allelic replacement mutant was significantly reduced in an IE model. These surface pili are biologically important and were found to be antigenic among humans during endocarditis. They are encoded by a ubiquitous operon in *E. faecalis* and may serve as a valuable immunological target for studies focusing on the prevention or treatment of this pathogen.

Our experience confirms that *E. faecalis*-induced infective endocarditis is the most severe and clinically challenging of all *E. faecalis*-related infections. The formation of endocardial vegetations is attributed to a biofilm formation on the heart valves, particularly in patients who do not respond positively to antibiotic treatment [62,63,64,65,71,75,80,81,109,114]. The evidence suggested by Nallapareddy and colleagues, who tested an unpiliated ebpA deletion mutant in the endocarditis model, further supports our experience [49]. The researchers noted that rats receiving a blend of wild-type *E. faecalis* OG1RF and its isogenic deletion mutant ebpA exhibited diverse levels of vegetation ebp. The percentages of ebpA were considerably lower in non-piliated mutants retrieved from the vegetation and kidneys 24 h after inoculation than in the percentages of wild-type mutants. These findings suggest that Ebp pili have a significant impact on this endovascular infection, similar to the function of biofilm in vitro.

Witten and colleagues [122] investigated the occurrence of allogeneic-tissue infection in patients with biological allografts implanted for indications other than endocarditis, as well as in patients with IE. The researchers used a parametric multiphase temporal decomposition non-proportional hazards and machine learning method analysis to assess the time-varying instantaneous risk of allograft infections in patients who underwent aortic-valve replacement with allogeneic tissue between 1987 and 2017. Over 2000 patients (n = 2042) underwent aortic-valve replacement using 2110 allografts for non-endocarditis indications (53%) and endocarditis indications (47%). PVE was present in 68% of cases, with recipients in the endocarditis-free group having a mean age of 52 ± 14 years and those with endocarditis having a mean age of 57 ± 15 years. The gram-positive cocci (GPC) were the most prevalent pathogens, particularly *Streptococcus viridans* (22%), *Staphylococcus aureus* (20%), *Enterococcus faecalis* (10%), and *group D streptococci* (11%), which are now recognised as *enterococcal* spp. Of these, S. aureus caused IE in 73% of injecting drug users and 63% of IE-causing pathogens were GPC biofilm-associated pili. The likelihood of allograft infection after 20 years was 14% in patients with IE and 5.6% in the cohort without IE. The utilisation of allogeneic tissue led to a significant enhancement in long-term survival, free from reinfection. It is noteworthy that most patients received an allograft for an unidentifiable cause and did not experience any recurrence of infection in the case of IE.

**Figure 10:**
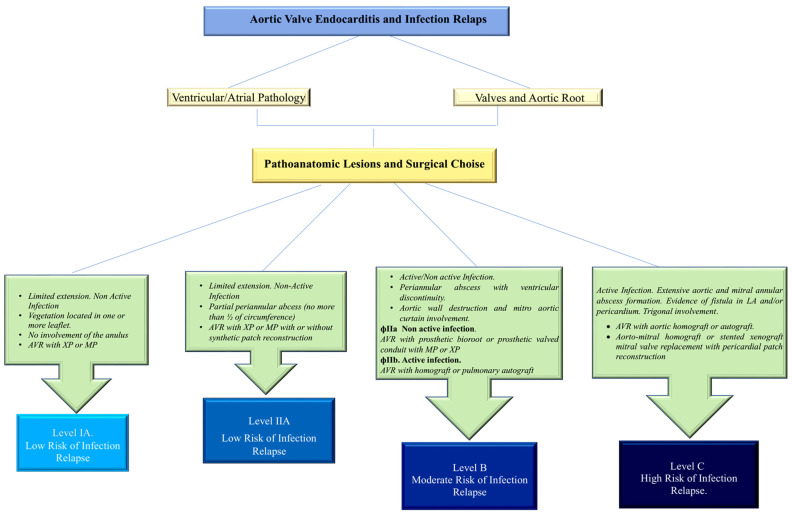
Algorithm to assess the risk of infection relapse due to GPC biofilm-associated pili. Risk categories are identified based on the anatomopathological characteristics of the infection, the magnitude of surgical demolition and reconstruction, and the materials used. Abbreviations: AVR, aortic-valve replacement; GPC, gram-positive cocci; MP, mechanical prosthesis. From Nappi et al. Refs. [62,63,64,65,68,71,78,79,80,81,109,114,125,126,140,141]. Delgado et al Eur Heart J. 2023 Aug 25, Ref. [68].

The study by Witten and colleagues [122] reports low infection rates in patients with and without endocarditis, supporting the ongoing use of allografts in modern times. This is especially true for treating invasive endocarditis of the aortic root with gram-positive cocci biofilm-associated pili, which confirms the effectiveness of biogene. The recent ESC guidelines recommend the use of allografts in experienced centres. Furthermore, they offer advantages in small aortic roots and demonstrate low reinfection rates, in addition to their effectiveness against root abscesses [68] (Figure 10).

## 8. Conclusions

IE after TAVI is an urgent medical condition that poses challenges in terms of diagnosis, management, and surgical intervention. The specific role and ideal timing of surgery are still debatable. However, adopting a multidisciplinary approach, prompt diagnosis, and early surgical intervention can enhance outcomes, as we observed in our case. Additionally, managing the increasing occurrence of multidrug-resistant strains of enterococci, including *Enterococcus faecalis*, often requires a multimodal management strategy. Consider using an allograft-valve substitute, particularly in cases of infective complications post-TAVI. Multiple studies have demonstrated the antimicrobial resistance of allograft valves, resulting in improved long-term outcomes.

## Data Availability

Not applicable.
